# MASLD Under the Umbrella of the Microbiota: A Narrative Review on Ecological Risk and Functional Transmissibility

**DOI:** 10.3390/jcm15041325

**Published:** 2026-02-07

**Authors:** Javier Crespo, Paula Argos Vélez, Marta Alonso-Peña, Lorena Cayón, Carolina Jiménez-González, Paula Iruzubieta

**Affiliations:** 1 School of Medicine, University of Cantabria, 39005 Santander, Spain; 2 Clinical and Translational Research in Digestive Diseases, Gastroenterology and Hepatology Department, Valdecilla Research Institute (IDIVAL), Marqués de Valdecilla University Hospital, 39008 Santander, Spain; paula.argos@idival.org (P.A.V.); marta.alonso@unican.es (M.A.-P.); lorena.cayon@idival.org (L.C.); carolina.jimenez@idival.org (C.J.-G.); 3 Departament of Anatomy and Cellular Biology, University of Cantabria, 39005 Santander, Spain

**Keywords:** MASLD, metabolic-hepatic risk, microbiota, modulability, ecosystem

## Abstract

Metabolic dysfunction-associated steatotic liver disease (MASLD) is the leading cause of chronic liver disease worldwide, distinguished by pronounced clinical heterogeneity and a frequent dissociation between metabolic risk factors and the degree of hepatic injury. These observations, together with the limited contribution of genetic heritability, have prompted a re-evaluation of the traditional conceptual framework of the disease. In this context, the question has emerged as to whether MASLD could be, at least in part, a transmissible condition. While there is no evidence to suggest that MASLD is contagious in humans, as no data support person-to-person transmission, gnotobiotic animal studies demonstrate that human gut microbiota can transfer susceptibility to steatosis, inflammation, and systemic metabolic disturbances through immunometabolic mechanisms, independent of host genetics. In parallel, human studies involving microbiota-targeted interventions support the concept that the gut ecosystem is a modifiable determinant of metabolic and hepatic phenotypes. Crucially, these findings do not imply natural transmission of disease, but rather underscore the functional plasticity of microbiota-host interactions. This narrative review integrates epidemiological, experimental, and clinical data to explore the hypothesis that MASLD may be functionally transmissible. MASLD is increasingly recognized as an eco-biological disease, where liver disease risk is not only shaped by host genetics and environment, but also by the ecological configuration and functional outputs of the gut microbiome. This perspective redefines disease susceptibility as, in part, context-dependent and microbiota-mediated, without implying infectiousness in the traditional sense.

## 1. Introduction

Metabolic dysfunction-associated steatotic liver disease (MASLD) has become, in less than three decades, the leading cause of chronic liver disease worldwide, with a prevalence exceeding 25% of the global population and a growing impact that has progressively displaced viral hepatitis as the dominant etiology in virtually all regions [1,2]. This expansion is not merely quantitative. In large global biopsy-based cohorts, advanced fibrosis is highly prevalent and independently associated with mortality and clinical events, confirming its role as the main prognostic determinant in MASLD [3]. In addition, several multinational projection models estimate that, in the absence of effective intervention, the clinical and economic burden of MASH will increase markedly over the next two decades, with substantial rises in direct healthcare costs and productivity losses [4].

The coexistence of clear familial aggregation with limited genetic heritability, together with marked interindividual heterogeneity and a frequent dissociation between classical metabolic burden and hepatic progression, has been consistently described in the literature. MASLD does not behave as a uniform entity, but rather as an emergent product of the dynamic interaction between genetic variants, environmental exposure, and metabolic profile. This interaction results in highly diverse clinical trajectories among apparently comparable individuals [5,6]. Particularly illustrative of this heterogeneity is the identification of metabolically distinct subtypes defined by metabolomic profiling, with divergent cardiovascular risk patterns that are independent of histological severity, reinforcing the need for systemic—rather than exclusively hepatic—risk stratification in clinical practice [7].

Within this context, asking whether MASLD could be, at least in part, a transmissible disease does not constitute a rhetorical provocation nor a concession to simplistic infectious models, as summarized in Table 1. Rather, the question arises from the need to reformulate the conceptual framework of the disease in light of accumulating evidence supporting a causal role of the intestinal microbiota in its natural history. Intestinal dysbiosis and functional alterations of the microbiome play a central role in MASLD pathogenesis, modulating metabolic, inflammatory, and fibrogenic processes beyond genetic predisposition and classical environmental factors [8,9]. Consistent with this framework, clinical cohorts have described differential fecal profiles between individuals with obesity with and without MASLD compared with controls, supporting the existence of microbial signatures associated with the phenotype, although not equivalent per se to disease transmission [10].

It is nevertheless essential to establish an unequivocal boundary from the outset. MASLD is not a contagious disease in humans. There are no epidemiological, clinical, or experimental data supporting person-to-person transmission in the classical sense. The specialized literature defines MASLD as a metabolic disease associated with obesity, diabetes, and metabolic syndrome, with no recognized routes of interpersonal contagion or infectious transmission mechanisms in humans [11]. This negative statement, however, does not exhaust the problem. Accumulating evidence, strengthened substantially in recent years, increasingly supports a more subtle and potentially transformative hypothesis: the existence of a functional transmissibility of metabolic–hepatic risk, mediated by microbial consortia and intestinal metabolic functions capable of modulating hepatic risk without implying infectious contagiousness. Intestinal dysbiosis and functional alterations of the microbiome are closely implicated in MASLD pathogenesis, regulating hepatic fat accumulation, inflammation, and fibrosis through the gut–liver axis and through microbially derived metabolites with immunometabolic effects [9,12].

## 2. MASLD Is Not Only About the Liver, but the Environment

The contemporary reformulation of MASLD has entailed a profound conceptual shift from a strictly hepatocentric view—focused on intrahepatocellular lipid accumulation—toward a systemic and multiorgan model, in which the liver acts primarily as a target organ of metabolic, inflammatory, and immunological dysfunctions that originate, fundamentally though not exclusively, outside the liver itself. This plausibility is not only conceptual but also anatomical: the liver is the first organ exposed to microbes, structural components, and intestinal metabolites, as it receives approximately 70% of its blood supply through portal circulation. This anatomical arrangement helps explain why alterations of the intestinal ecosystem can be directly translated into hepatic immunometabolic signaling [13]. This change in perspective is not merely terminological; it reflects growing evidence that the natural history of MASLD is determined by the integration of signals arising from adipose tissue, the intestine, the innate immune system, and the global metabolic environment. This integration accounts for both the marked phenotypic heterogeneity and the frequent dissociation between classical metabolic burden and hepatic progression observed in clinical practice [14].

Within this expanded framework, the intestinal microbiota is no longer regarded as an epiphenomenon associated with obesity or diet, but has consolidated its role as a relevant causal actor, embedded within complex pathogenic networks that coordinately modulate inflammation, metabolism, and hepatic fibrogenesis. Crucially, this paradigm has been further extended by the demonstration that the liver is not a sterile compartment. Experimental and translational studies have identified the presence of an intrahepatic microbiome, quantitatively sparse but compositionally distinct from the intestinal microbiota, selectively populated from the gut and capable of modulating local immune responses. These findings challenge the classical model of the liver as a bacteriologically isolated organ [15] as indicated in Table 2.

Consistent with this view, MASLD-associated dysbiosis is not limited to taxonomic shifts but involves a profound functional reprogramming of the microbiome, with alterations in key metabolic pathways. These include bile acid signaling (FXR–FGF19, TGR5), endogenous ethanol production, choline metabolism, and the generation of pro-inflammatory microbial metabolites capable of activating hepatic innate immunity [23]. Collectively, these pathogenic drivers and potential intervention targets have been reviewed in an integrative manner focused on mechanisms and therapeutic opportunities in MASLD [24].

At this point, it is essential to clarify that the term “microbiome” does not equate to a simple list of taxa. It also encompasses its true “theater of activity”: microbial structural components, local environmental conditions, and a broad repertoire of metabolites and signals. This reinforces the notion that the biologically relevant unit of risk is not always the microbial species itself, but rather the function that the ecosystem deploys within a given context [13].

In 2025, Lau and colleagues describe MASLD, MASH, and hepatocellular carcinoma as entities profoundly shaped by the dynamic interaction among the bacteriome, virome, and mycobiome, emphasizing that the intestinal microbial ecosystem acts simultaneously as a context-dependent pathogenic driver, a modulator of systemic inflammation, and an emerging therapeutic target [25]. Complementarily, Schnabl and Brenner integrate these concepts into a mechanistic model in which disruption of the intestinal barrier, translocation of microbial products, and signaling mediated by microbiome-derived metabolites constitute central causal axes in the progression from steatosis to hepatic inflammation and fibrosis [20].

This body of evidence therefore compels the conceptualization of MASLD as a genuinely eco-biological disease, in which the hepatic phenotype emerges from the non-linear interaction between the human genome, intestinal microbial ecosystems, and the environmental exposome, including diet, medications, circadian rhythms, and the social environment. Although the term “eco-biological” does not represent a formal nosological category, it arises from the convergence of established theoretical frameworks: eco-social epidemiology and the concept of embodiment—the process by which social and environmental conditions are biologically incorporated [26]; the exposome as an indispensable complement to genetics [27]; and systems medicine applied to complex diseases [28].

Within this context, an emerging element of particular relevance is intestinal chronobiology. The circadian clock of the intestinal epithelium regulates the composition, function, and metabolites of the microbiome, and the transfer of microbiota from animals with disrupted intestinal clocks alters gene expression and immunometabolic homeostasis in recipient hosts, demonstrating an additional form of non-infectious functional transfer [29].

## 3. Experimental Evidence Supporting How Hepatic Risk Becomes Transferable

The most robust support for the notion of the functional transmissibility of metabolic–hepatic risk derives from animal experimentation, particularly from gnotobiotic models and fecal microbiota transplantation (FMT), in which genetic transfer can be clearly dissociated, at a methodological level, from ecological transfer (Table 3). The central concept emerging from this body of evidence is not that MASLD is “contagious,” but that determinant components of the metabolic–hepatic phenotype can be transferred when an intestinal ecosystem capable of reprogramming host metabolism and immunity is transferred. In this regard, a framework that is particularly coherent with the concept of functional transmissibility is that of microbiome fermentative pathways: imbalances in metabolic routes—for example, predominance of alcoholic fermentation over short-chain fatty acid–producing pathways—may sustain an eco-metabolic vicious circle that favors progression toward MASH [17,30].

A foundational milestone in this field was the study by Le Roy and colleagues, which elegantly demonstrated that the intestinal microbiota is sufficient to determine susceptibility to hepatic steatosis in mice. Through cross-fecal transplants between “MASLD-prone” and “MASLD-resistant” animals, the authors observed that recipients acquired metabolic and hepatic traits consistent with the donor phenotype independently of diet, thereby establishing a key principle: the risk of liver disease can be transferred without genetic transfer [31]. This principle is further reinforced when microbial transfer modulates not only steatosis but also more complex inflammatory and immunometabolic profiles. In a paradigmatic study, Henao-Mejía et al. showed that inflammasome deficiency (NLRP6/NLRP3) is associated with a dysbiosis capable of exacerbating obesity, metabolic syndrome, and progression of MASLD/MASH; critically, this phenotype behaves as microbiota-transferable in experimental models, establishing a causal link between microbial ecology, innate immunity, and liver injury [32].

Beyond the intestine, it has also been demonstrated that microbes present in the liver can directly program hepatic immunity. In murine models and humans, bacterial species enriched in the intrahepatic compartment present glycosphingolipids to Natural Killers T (NKT) cells, activating CCL5-mediated signaling and promoting the expansion and activation of hepatic leukocytes, thereby establishing a microbe–immunity axis with pathogenic potential in metabolic liver diseases [33].

Functional transmissibility does not necessarily require transplantation of an entire microbial community. High-causal-strength studies have shown that specific microbial functions, carried by defined strains, are sufficient to induce hepatic phenotypes. A reference example is the identification of Klebsiella pneumoniae strains with a high capacity for endogenous ethanol production: human clinical isolates, administered orally, induced a fatty liver phenotype in mice, establishing a direct connection between a defined microbial function—endogenous alcohol production—and the development of steatosis [16]. Convergently, classical work in obesity and insulin resistance, major determinants of MASLD risk, provides indirect but highly relevant causal evidence. Ridaura et al. demonstrated that microbiota from human twin pairs discordant for obesity transfers to gnotobiotic recipients a metabolic phenotype consistent with the donor, underscoring that systemic traits can be modulated by microbial communities [34]. Even more incisively, Fei and Zhao isolated a human intestinal bacterium (Enterobacter cloacae B29) whose colonization of germ-free mice induced obesity and insulin resistance, demonstrating that a specific microbial component can activate inflammatory–metabolic pathways with causal capacity [35].

Experimental evidence further shows that the microbiota can transfer benefit, not only risk, reinforcing its role as a modulable functional vector. Of particular relevance, functional transfer can be protective: improvement of hepatocellular mitochondrial function generates a specific microbial signature that, when transferred to germ-free mice, delays progression of MASH, demonstrating that the intestinal ecosystem can convey metabolic resilience in addition to risk [36]. Lei et al. showed that transfer of fecal microbiota from humans treated with disulfiram to germ-free mice improved MASH phenotypes in recipients, supporting the concept that therapeutically induced microbial functions can be experimentally transferred [37]. Concordantly, other studies have shown that transplantation of “healthy” microbiota can attenuate diet-induced steatohepatitis [38].

A particularly relevant nuance for the functional transmissibility hypothesis is that, in some models, human microbiota induces steatosis even in the absence of an obesogenic diet. Wang et al. demonstrated that microbiota from a genetically obese human donor was capable of inducing steatosis in germ-free mice fed a standard diet, providing an additional example of diet-independent functional transfer [39].

Taken together, these experimental models establish three coherent propositions. First, that metabolic–hepatic risk can “travel” with microbial communities or with specific microbial functions, without genetic transfer. Second, that the observed transmissibility is functional and context-dependent, and not equivalent to classical infection. Third, that the microbiota operates through plausible metabolic and immunological pathways—such as metabolic endotoxemia, diverse microbial metabolites, a toxic bile acid profile, and increased intestinal permeability—constituting a causally demonstrable mechanism. The mechanistic plausibility of metabolic endotoxemia has been further reinforced by single-cell transcriptomic studies showing that exposure to microbial products induces a zonal pro-inflammatory reprogramming of periportal hepatocytes, with macrophage recruitment via the CCL2–CCR2 axis and secondary suppression of adaptive immunity, thereby establishing a causal link between systemic microbial signaling and liver injury [40]. In humans with MASLD, detection of circulating bacterial antigens is likewise associated with increased pro-inflammatory cytokines and activation of TLR pathways, even independently of body mass index, supporting a systemic mechanism compatible with low-grade endotoxemia [21].

## 4. Clinical Evidence in Humans That Demonstrates Modulability Without Contagiousness

In humans, the evidence linking the intestinal microbiota to metabolic–hepatic risk necessarily takes a different form from experimental models and requires a particularly cautious interpretation. Unlike animal models, clinical research cannot—and should not—explore “spontaneous transmission” of pathological phenotypes through uncontrolled microbial exposure. Consequently, available knowledge is based on deliberate therapeutic interventions (fecal microbiota transplantation, diet, prebiotics/probiotics, or other modulation strategies), designed to interrogate the modulability of metabolic traits and, when feasible, hepatic outcomes, under controlled conditions and with clinical follow-up.

The first clinical milestone that opened this field was the trial by Vrieze et al., which demonstrated that fecal microbiota transplantation (FMT) from lean donors significantly improves insulin sensitivity in individuals with metabolic syndrome [41]. This study established, for the first time in humans, a core concept for this conceptual framework: systemic metabolic traits can be functionally transferred via the microbiota without implying disease transmission. Subsequent studies confirmed and refined these findings, showing that the magnitude of the effect depends on the recipient’s baseline microbiome composition and is associated with changes in short-chain fatty acid production, bile acid metabolism, and low-grade inflammatory signaling [42]. Available meta-analyses reinforce this cautious interpretation: in randomized trials, FMT from lean donors produces early and modest improvements in metabolic syndrome parameters (insulin sensitivity, HDL), without sustained effects on body weight and without induction of de novo disease. This underscores that human microbial transfer modulates functional traits but does not transmit established metabolic pathology [43].

In the specific field of hepatology, early clinical trials of FMT in MASLD yielded heterogeneous but conceptually informative results. Controlled studies suggested that microbiota manipulation can improve intestinal permeability, reduce metabolic endotoxemia, and modulate systemic inflammatory markers, even when changes in hepatic steatosis were modest [44]. Part of this heterogeneity reflects not only biological variability, but also measurement and design limitations: demonstrating benefit on “hard” hepatic outcomes requires prolonged follow-up, and many trials rely on biopsy-based intermediate endpoints, which are subject to sampling error and interobserver variability. Accordingly, consortia such as LITMUS have emphasized the qualification of non-invasive biomarkers to improve efficiency, patient selection, and comparability of results [45]. Consistent with this interpretation, recent reviews emphasize that clinical response to dietary, prebiotic, or probiotic interventions in MASLD critically depends on the prior functional state of the microbiome—including its capacity to produce short-chain fatty acids, modulate bile acids, and limit endotoxemia—reinforcing the need for eco-biological precision medicine approaches [46].

To date, a particularly relevant clinical contribution, because of its methodological ambition, is the randomized trial published in 2025 by Groenewegen et al., which evaluated consecutive fecal microbiota transplants (allogeneic versus autologous) in patients with MASLD, using MRI-PDFF as the primary endpoint [47]. Beyond its specific results, the study represents a turning point by integrating (i) a quantitative hepatic outcome, (ii) an ecologically plausible intervention design, and (iii) longitudinal metagenomic analyses. Taken together, it reinforces the concept that the microbiota constitutes an actionable target in MASLD. The rationale, indications, and current limitations of FMT as a strategy in MASLD have been synthesized in recent reviews, which emphasize that the clinical objective is functional modulation—and not disease transfer [48].

Complementary to fecal transplantation-based interventions, incretin pharmacology provides a particularly robust example of the clinical modulability of the intestinal ecosystem in humans. Recent preclinical and clinical evidence shows that GLP-1 receptor agonists and dual or triple agonists induce a reproducible functional reprogramming of the intestinal microbiota, mediated by changes in motility, bile acid metabolism, and intestinal permeability, contributing to improvement of the metabolic and hepatic phenotype in MASLD. These effects, documented through longitudinal metagenomic and metabolomic approaches, reinforce the notion that the microbiota acts as a therapeutic modulator of metabolic–hepatic risk, without implying interpersonal transmission of disease in any case [20,49,50].

MASLD treatment with SGLT2 or RAS inhibitors, vitamin E, or statins affects the liver–gut axis and influences microbiota in an indirect manner, altering weight, nutrient flow, bile acids and inflammation [51]. SGLT2 inhibitors improve MASLD by enhancing fatty-acid β-oxidation, reducing de novo lipogenesis, and attenuating inflammation, oxidative stress, and fibrosis; and, additionally, modulating gut microbiota [52]. RAS inhibitors modulate barrier function and inflammation, reducing fibrosis progression and improving cardiometabolic outcomes. However, whether this is somehow mediated by microbiota remains unproven [51]. Vitamin E has an antioxidant role in reducing hepatic oxidative stress and inflammation, although how microbiota are involved remains controversial [53]. Statins, besides their role in reducing cardiovascular risk, have pleiotropic anti-inflammatory effects and can alter bile-acid synthesis and FXR–TGR5 signaling, which are tightly coupled to microbial composition [51].

It is nevertheless essential to underscore the interpretative boundary that underpins this thesis: none of these trials evaluate natural transmission of disease between individuals. These are controlled medical interventions, conducted in specific clinical contexts, with recipient selection and protocolized follow-up. Even in these scenarios of deliberate transfer, strain engraftment is variable, dependent on the recipient’s prior ecosystem, and not sufficient by itself to induce de novo hepatic disease in the absence of a susceptible metabolic substrate. This interpretation is consistent with recent clinical syntheses. The review on MASLD in adults published in JAMA in 2025 integrates the microbiota as a relevant modulator of risk and hepatic progression—in its interaction with obesity, diabetes, genetics, and the exposome—without suggesting mechanisms of interpersonal transmission [54]. A recent review further reinforces this position, emphasizing that the clinical relevance of the microbiota lies in its therapeutic and preventive potential, not in its role as an infectious vector [14,23].

## 5. Sharing Microbiota Is Not Equivalent to Sharing Disease

A necessary starting point for rigorously addressing the relationship between the intestinal microbiota and MASLD is to dismantle a recurrent confusion: cohabitation-induced microbial convergence is not equivalent to disease transmission. It is well established that people who share a physical environment and daily habits tend to exhibit more similar intestinal microbiotas than non-cohabiting individuals, a phenomenon consistently described as the “household effect.” In a seminal study, Song et al. showed that members of the same household—including humans and domestic animals—share relevant components of their gut microbiota, underscoring the weight of the shared environment on microbial composition [55]. Concordantly, Dill-McFarland et al. confirmed that social proximity and cohabitation explain a significant proportion of interindividual variability in the human microbiome, independently of genetic relatedness [56]. These findings were subsequently reinforced by larger-scale analyses that positioned the environment as the dominant determinant of the human microbiome: the study by Rothschild et al. showed that host genetics account for only a minor fraction of microbial variability, whereas environmental factors such as diet, lifestyle, and surroundings exert a substantially greater impact [57].

Within the same logic, the intestinal virome should be interpreted primarily as a modulator of the microbial ecosystem rather than as an infectious agent: bacteriophages influence bacterial population dynamics, the transfer of metabolic functions, and liver inflammation associated with MASH, without evidence of direct viral transmission of liver disease between humans [58]. However, the fact that cohabitation induces microbial convergence does not imply MASLD contagiousness. Despite the robustness of these ecological data, there is no longitudinal evidence causally linking microbial similarity among cohabitants to the development of MASLD once classical metabolic determinants—such as obesity, insulin resistance, diet, physical activity, or alcohol consumption—are adequately controlled; these determinants also maintain complex and clinically relevant interactions. At this point, the interaction between alcohol and metabolic liver disease warrants specific mention given its relevance and clinical ambivalence, including the debate on the impact of low-to-moderate intake and the synergy with obesity/diabetes [59].

In particular, no study has demonstrated that acquisition of MASLD-associated microbial signatures in one cohabitant temporally precedes the development of hepatic steatosis or other objective hepatic outcomes in the other, once adjustment is appropriately made for shared exposures (diet, activity, medications, alcohol, sleep deprivation, socioeconomic status, and other exposome components). This distinction becomes even clearer when contexts of deliberate and massive microbial transfer are examined: even in clinical scenarios such as fecal microbiota transplantation, the engraftment of specific strains is heterogeneous, highly dependent on the recipient’s pre-existing intestinal ecology, and mediated by complex microbe–microbe interactions [60,61]. Particularly revealing clinical studies integrating metagenomics and metabolomics have shown that fecal concentrations of butyrate and deoxycholic acid predict short-term mortality in patients with advanced liver disease more strongly than the abundance of specific bacterial species, confirming that the clinically relevant determinant is the ecosystem’s metabolic function, not its taxonomic composition [62].

**Figure 1:**
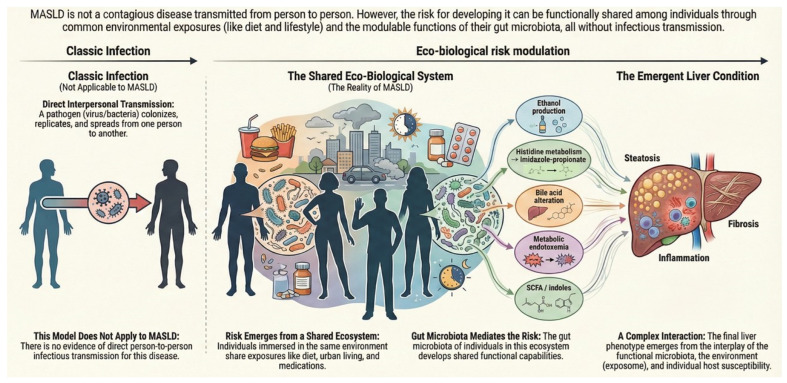
Eco-biological framework modulates metabolic–hepatic risk.

Moreover, experimental and translational evidence converges on an interpretative principle that should be stated precisely: the microbiota does not behave as an autonomous pathogen, but as a conditioned modulator of metabolic–hepatic risk. Microbial transfer can modulate the emergence of steatosis under dietary challenge in animal models [31], and transferable metabolic phenotypes tend to be diet-dependent and reversible [34]. In humans, microbiota-based interventions have been shown to modify intermediate metabolic traits without inducing de novo hepatic disease [41]. In strictly defined terms, therefore, the microbiota can be shared; MASLD cannot (Figure 1).

MASLD does not follow the classic infection pattern, as there is no evidence of direct person-to-person transmission of the disease. In fact, the risk of developing MASLD is influenced by ecological and biological factors. Individuals in the same environment (shared ecosystem) have similar exposures, such as diet, urban living and medication. The gut microbiota mediates this risk through the development of shared functional capabilities, including ethanol production, histidine metabolism, bile acid alteration, metabolic endotoxemia, and the metabolism of short-chain fatty acids (SCFAs) and indoles. The resulting liver condition emerges from the complex interplay between functional microbiota, the environment (exposome) and individual host susceptibility.

## 6. From Clinical Modulability to Functional Transmissibility of Metabolic–Hepatic Risk

From a systems biology perspective, functional transmissibility of risk should be understood as a phenomenon mediated by local barriers. Epithelial interfaces actively regulate communication between the microbiota and the host through metabolites, proteins, and small molecules, such that alterations in these barriers can translate into systemic metabolic and immune effects without requiring infectious transmission [63]. This conceptualization is particularly relevant in MASLD, where the disease is not transmissible in the classical infectious sense, yet the notion of functional transmissibility of metabolic–hepatic risk is emerging with increasing clarity.

This hypothesis is articulated through the convergence of three independent but complementary lines of evidence:

(i) Experimental demonstration that relevant metabolic functions can be transferred via the microbiota without genetic transfer;

(ii) Clinical evidence that these traits are modulable in humans through controlled interventions; and

(iii) The recent development of methodological tools capable of documenting, with high resolution, the transfer, persistence, and functional activity of specific microorganisms and microbial functions in the human intestine.

Within this framework, functional transmissibility of metabolic–hepatic risk extends even to the prenatal period. Twin studies have shown that an unfavorable intrauterine environment predominates over genetics in early shaping of the microbiome and intestinal metabolome, generating dysbiosis and metabolic alterations detectable from birth and associated with adverse metabolic outcomes in mid-term follow-up, without implying transmission of liver disease per se [64]. A paradigmatic example of metabolite-mediated functional transmissibility is indole-3-propionate (IPA), a microbial tryptophan derivative. Population-based studies integrating longitudinal microbiome, diet, and metabolomics data have demonstrated that higher circulating IPA concentrations—determined by the interaction between specific bacterial species and high fiber intake—are robustly associated with lower risk of type 2 diabetes, underscoring that what is functionally transmitted is not a specific microorganism, but an emergent metabolic capacity of the ecosystem [19].

An additional example that is particularly illustrative of this logic of metabolite-mediated functional transmissibility is microbial histidine metabolism. Recent studies have shown that certain functional configurations of the intestinal microbiome convert dietary histidine into imidazole propionate, a metabolite capable of directly interfering with insulin signaling through activation of mTORC1 and inducing insulin resistance in humans. Of relevance to the conceptual framework proposed here, imidazole propionate production does not depend on the presence of a universally pathogenic taxon, but on the emergence of functional microbial consortia favored by shared dietary and metabolic contexts. This metabolite has been associated with obesity and metabolic dysfunction in humans and constitutes a paradigmatic example of how specific microbial functions—rather than discrete microorganisms—can convey cardiometabolic and hepatic risk without requiring infectious transmission or stable colonization. In the context of MASLD, this mechanism provides an additional pathway, complementary to classical metabolic endotoxemia, through which the intestinal ecosystem can functionally and contextually modulate hepatic insulin resistance and disease progression [18,65,66,67,68]. Consistent with this view, metagenomic analyses have shown that changes in species abundance do not necessarily correspond to variations in key metabolic genes, supporting a gene-centric approach to the microbiome in which function can be modified without major taxonomic shifts [48].

A decisive inflection point in the empirical consolidation of this concept has been the ability to discriminate among environmental coexistence, transient colonization, and stable engraftment at the strain level. The seminal study by Smillie et al. established that engraftment after fecal microbiota transplantation can be precisely tracked at the strain level and depends on recipient-specific ecological determinants, including baseline abundance and the phylogeny of pre-existing taxa [60]. This methodological framework has been expanded through metagenomic genome reconstruction, probabilistic source tracking, and longitudinal multi-omics analyses [69,70]. On this basis, more recent studies have demonstrated that engraftment is not a passive process, but the result of highly structured microbe–microbe interactions. Work by Chen et al. showed that effective transfer after FMT is conditioned by networks of competition and cooperation between donor and recipient strains, determining functional persistence and explaining the marked interindividual variability observed in clinical trials [61].

Of particular relevance to the concept of functional transmissibility is that such transfer can occur even in the absence of stable donor colonization. It has been demonstrated that mobile genetic elements of the maternal microbiome can shape assembly and metabolic potential of the infant microbiome through horizontal gene transfer—including mediation by phages and other components of the mobilome—introducing an intergenerational pathway of functional transfer without classical vertical transmission of species [71]. This framework extends to the perinatal period, where maternal–infant microbial transmission during pregnancy, delivery, and lactation contributes to early immunometabolic imprinting, with persistent effects on the risk of obesity, diabetes, and metabolic disease in adult life, without implying transmission of liver disease per se [72].

In parallel, metabolic hepatology has incorporated these technical capabilities into quantitative hepatic outcomes. The randomized clinical trial by Groenewegen et al. represents the most advanced example to date: through consecutive allogeneic versus autologous fecal microbiota transplants and the use of MRI-PDFF as the primary endpoint, the study demonstrated that targeted manipulation of the microbial ecosystem can objectively modify hepatic steatosis in patients with MASLD [47]. These observations integrate with recent mechanistic models in which disruption of the intestinal barrier, translocation of microbial products, metabolite-mediated signaling, and activation of innate immunity constitute central causal axes of MASLD progression [20]. Reviews in Nature Reviews Gastroenterology & Hepatology reinforce that MASLD-associated dysbiosis involves a functional reprogramming of the microbiome that is sensitive to shared environmental factors [14,23,25].

**Figure 2:**
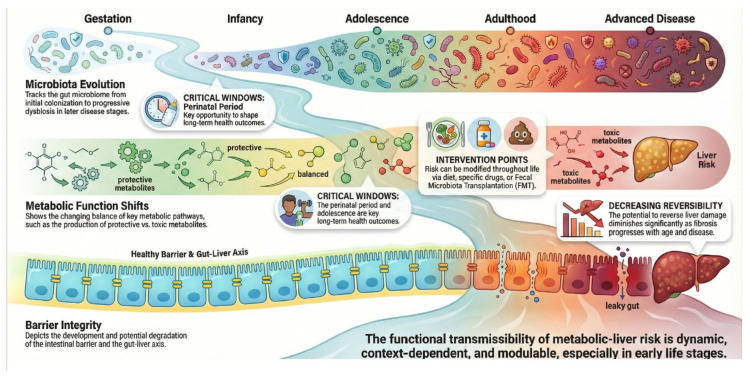
Functional transfer of metabolic liver disease risk across the lifespan.

A particularly illustrative example of shared environmental modulability is alcohol. Experimental evidence indicates that ethanol does not act as a direct microbial substrate; rather, its hepatic metabolism increases systemic acetate, which functionally reprograms the intestinal microbiota, reproducing dysbiotic signatures similar to those induced by chronic alcohol consumption without requiring direct luminal exposure [22]. Taken together, these data consolidate a coherent framework in which metabolic–hepatic risk can be shared and functionally modulated through the microbial ecosystem and its products, without implying infectious contagiousness or transmission of liver disease in the classical sense (Figure 2).

Epithelial interfaces actively regulate communication between the microbiota and the host through metabolites, proteins and small molecules. Alterations to these barriers can therefore lead to systemic metabolic and immune effects, resulting in the progression of MASLD. The microbiome evolves throughout the lifespan, from initial colonization during gestation through to infancy, adolescence, and adulthood, leading to advanced disease stages. Metabolic function shifts, changing the balance of key metabolic pathways from protective to one that favors toxic metabolites and liver disease risk. There are critical windows during the perinatal period and adolescence that are key to long-term health outcomes. However, risk can be modified throughout life via intervention points, such as diet, specific drugs, or fecal microbiota transplantation (FMT). At the stage of disease, the integrity of the barriers are impaired, including potential for degradation of the intestinal barrier and the gut–liver axis. The ability to reverse liver damage diminishes significantly as fibrosis progresses with age and disease. The result is a functional transmissibility of metabolic liver disease risk that is dynamic, context-dependent, and modulable, especially in early life stages.

## 7. Implications for Public Health: From the Non-Contagious Individual to Population-Level Ecosystem Risk

The evidence reviewed necessitates a clarification with direct public-health consequences: the absence of interpersonal transmission of MASLD does not invalidate, but rather reinforces, the need for a population-based approach, as summarized in Table 4. The distinction between infectious contagiousness and functional transmissibility of risk is critical. Whereas the former lacks epidemiological, clinical, or experimental support, the latter provides a coherent conceptual framework for understanding how shared metabolic and microbial environments can modulate hepatic risk at a collective scale. In this sense, it has been explicitly proposed to prioritize MASLD as an indicator and strategic target of global cardiometabolic health, comparable in urgency to other recent public-health agendas [73].

From a longitudinal perspective, MASLD is embedded within an eco-biological continuum that connects metabolic steatosis with cirrhosis and its complications. Along this trajectory, intestinal dysbiosis, epithelial barrier dysfunction, and translocation of microbial products progressively intensify, modulating systemic inflammation and the risk of hepatic decompensation without requiring infectious transmission between individuals [74,75]. This framework is further reinforced by evidence from cirrhosis, where dysbiosis and barrier dysfunction not only accompany disease progression but actively modulate the risk of decompensation and multiorgan failure, even in the absence of an identifiable infectious trigger. These observations underscore eco-microbial continuity between MASLD, cirrhosis, and acute clinical events [76].

This approach aligns with classical principles of population epidemiology, particularly the concept articulated by Geoffrey Rose, according to which the burden of disease in a population depends primarily on modest but sustained shifts in the population mean risk, rather than on the aggregation of high-risk individuals alone [77]. MASLD, given its high prevalence and its close linkage to shared environmental determinants, constitutes a paradigmatic example of this principle. From this perspective, the intestinal microbiota can be understood as a biological interface between structural determinants of health—food systems, urbanization, socioeconomic inequality, pharmaceutical and antibiotic exposure—and the individual metabolic–hepatic phenotype. The exposome concept provides the theoretical framework to integrate these cumulative exposures across the life course [27], while eco-social epidemiology emphasizes how biological processes embody social and environmental conditions [26].

Applied to MASLD, this framework implies that prevention cannot be confined to individual behavior alone. Authoritative reviews have emphasized that MASLD-associated dysbiosis reflects a functional reprogramming sensitive to shared environmental factors, rather than a stochastic, purely individual process [14,20,23]. Beyond the biomedical domain, social and commercial determinants of health, as well as their associated stigma, exert a decisive influence on risk, diagnosis, and clinical outcomes in MASLD, reinforcing the need for integrated and multisectoral approaches [78].

Consequently, the hypothesis of functional transmissibility of risk does not imply measures typical of infectious disease control, but rather structural interventions: healthy food policies, active urban design, regulation of antibiotic use, reduction of social inequalities, and population-level preventive strategies coherent with precision preventive medicine [28]. In this direction, the integration of microbiome multi-omics and artificial intelligence has been proposed to develop personalized, scalable, and accessible nutrition strategies—including chatbots and digital tools—as a potential pathway to democratize lifestyle interventions in MASLD [79]. Within this context, the Western diet can be understood as a population-level ecological vector capable of inducing dysbiosis and chronic inflammatory states that are functionally transmissible beyond individual disease, a concept extensively developed in the field of intestinal inflammation and extrapolatable to metabolic liver disease [80]. Complementarily, from a population perspective, fermented foods illustrate how repeated, culturally integrated, and safe microbial exposure can modulate microbiota and metabolic function without infectious risk. These foods act as reservoirs of microorganisms, metabolites, and bioactive compounds that reinforce the intestinal barrier and attenuate systemic inflammation, providing a practical model of ecological risk modulation [81].

## 8. Conclusions

Accumulated evidence from epidemiological, clinical, and experimental studies shows unequivocally that MASLD is not transmitted from person to person in the traditional infectious sense. The critical integration of the data reviewed in this work converges on a more complex and epistemologically demanding model: metabolic–hepatic risk is ecologically modulable, dependent on microbial functions and shared exposures, and potentially transferable at a functional level.

Experimental models consistently demonstrate that microbial communities and specific metabolic functions can reprogram host metabolism and immunity, transferring susceptibility or protection against steatosis and its progression without genetic transfer. The liver acts primarily as a target organ of dysfunctions generated in interconnected biological systems—intestine, adipose tissue, innate immunity, and the metabolic environment—accounting for marked interindividual heterogeneity, familial aggregation not explained by classical genetics, and the dissociation frequently observed in clinical practice between apparent metabolic burden and hepatic progression. Furthermore, accumulated evidence indicates that the microbiome’s impact on the liver begins very early in life and the risk of developing several diseases, including MASLD, can be shared, modulated, or attenuated at the population level without the disease being transmissible in an infectious sense.

In conclusion, MASLD is not a contagious disease, but it is not a purely individual disease either. It is an eco-biological and ecosystem disease in which risk can be shared without the disease being shared, and in which prevention and treatment require moving beyond hepatocentric and purely behavioral approaches to incorporate interventions directed at the biological and social systems that determine its clinical expression. This conceptual distinction does not close the debate; it places it on empirically addressable and clinically relevant ground, aligned with contemporary challenges in metabolic hepatology and global public health.

## Figures and Tables

**Table 1:** Infectious contagiousness versus functional transmissibility of metabolic–hepatic risk.

Dimension	Infectious Contagiousness	Functional Transmissibility of Metabolic–Hepatic Risk
Transferred biological unit	Identifiable pathogen (virus, bacterium, parasite)	Metabolic functions, microbial consortia, metabolites
Primary mechanism	Infection, replication, and dissemination	Immunometabolic and ecological modulation
Need for stable colonization	Yes, essential requirement	Not necessarily; can be transient or functional
Dependence on host context	Limited	Very high (diet, exposome, genetics, metabolic state)
Human epidemiological evidence in MASLD	Non-existent	Indirect, functional, and contextual
Experimental evidence	Not available	Robust (gnotobiotic models, FMT, microbial functions)
Reversibility of the phenomenon	Low	High and environment-dependent
Risk of interpersonal transmission	Yes	No
Clinical implications	Isolation, contagion control	Population prevention, ecological intervention
Public-health relevance	Not applicable to MASLD	High

FMT, fecal microbiota transplantation; MASLD, metabolic-associated steatotic liver disease.

**Table 2:** Microbial functions with capacity for functional transmissibility of metabolic–hepatic risk.

Dominant Microbial Function	Main Metabolite/Pathway	Metabolic–Hepatic Effect	Type of Evidence	Representative References
Endogenous ethanol production	Ethanol	Hepatic steatosis and inflammation	Experimental	[16]
Dysbiotic fermentation	↓ SCFA/↑ alcohols	Progression to MASH	Experimental	[17]
Histidine metabolism	Imidazole propionate	Insulin resistance, metabolic dysfunction	Human + experimental	[18]
Tryptophan metabolism	Indole-3-propionate	Cardiometabolic protection	Human	[19]
Bile acid metabolism	Secondary bile acids	Inflammation, FXR/TGR5 signaling	Human + experimental	[20]
Metabolic endotoxemia	LPS	Hepatic immune activation	Human	[21]
Acetate-driven reprogramming	Systemic acetate	Alcohol-like dysbiosis	Experimental	[22]

↓, decrease; ↑, increase; FXR, Farnesoid X Receptor; LPS, Lipopolysaccharide; MASH, metabolic-associated steatohepatitis; TGR5, Takeda G-protein-coupled receptor 5; SCFA, short-chain fatty acid.

**Table 3:** Levels of evidence on microbiota and MASLD: scope and interpretation.

Level of Evidence	Study Design	What It Shows	What It Does Not Show
Experimental	Gnotobiotic models, animal FMT	Functional causality	Direct population relevance
Translational	Human FMT	Metabolic modifiability	Natural transmission
Observational	Clinical cohorts	Consistent association	Direct causality
Multi-omics	Metagenomics + metabolomics	Biologically relevant function	Unique taxonomic origin
Epidemiological	Cohabitation, households	Environmental convergence	Contagiousness
Interventional	Diet, prebiotics	Functional reversibility	Definitive cure

FMT, Fecal microbiota transplantation.

**Table 4:** Clinical and public-health implications of the functional transmissibility of risk.

Level	Key Implications
Individual	MASLD is not transmissible between people
Clinical	The microbiota enables risk stratification and risk modulation
Preventive	Early dietary and ecological interventions
Population	Food environments as risk modulators
Health-policy	Prioritize MASLD as an indicator of cardiometabolic health
Ethical–social	Shift focus from individual stigma to structural determinants
Research	Functional targets > taxonomic targets

## Data Availability

No new data were created or analyzed in this study.
